# Supplementary figures and images for: Factors associated with the leftover rate of side dishes in Japanese school lunches
Source: PLoS One. 2024 Feb 26;19(2):e0298691. doi: 10.1371/journal.pone.0298691 (PMC10896521; doi:10.1371/journal.pone.0298691)

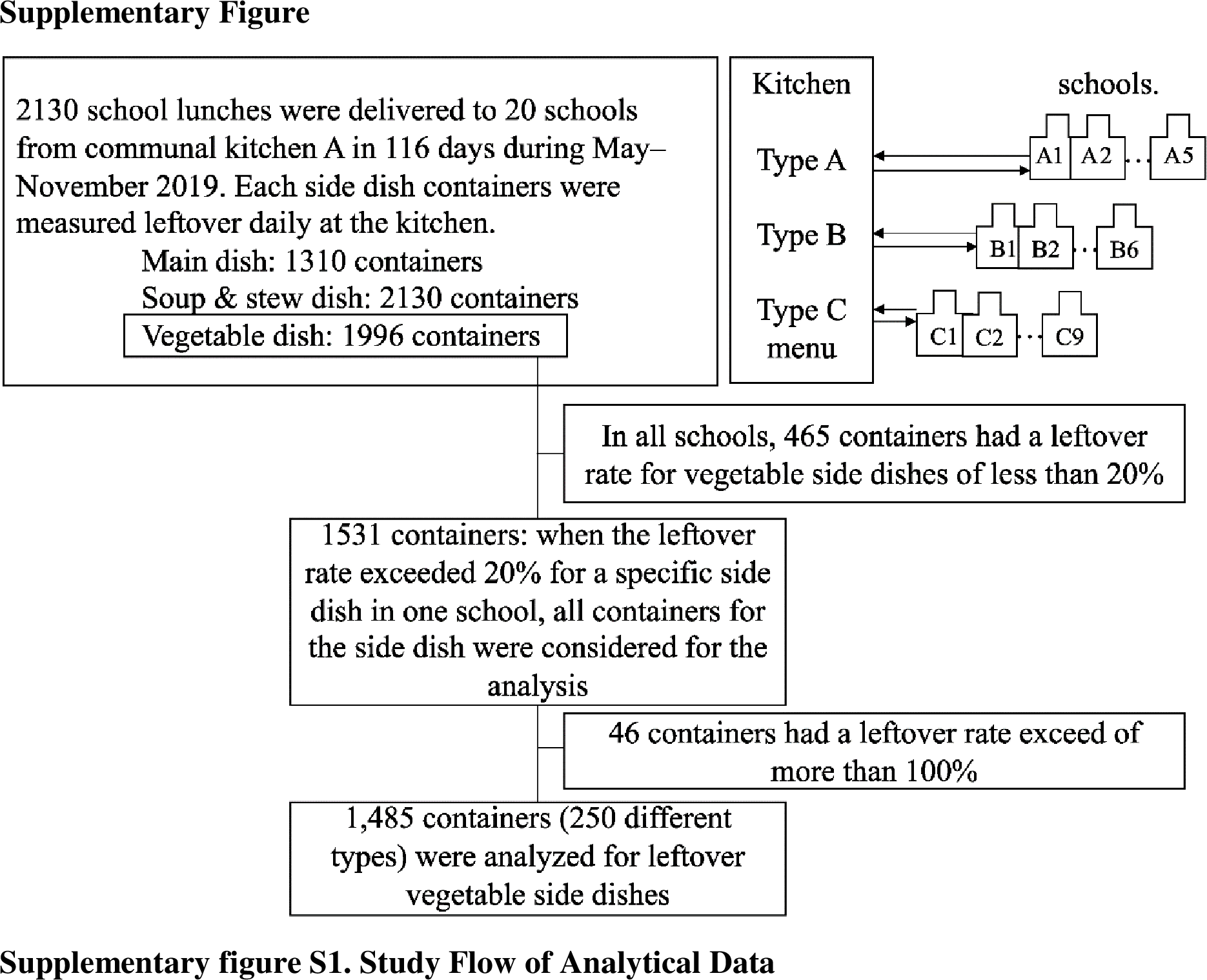

Supplement: S1 Fig — (TIF) [file pone.0298691.s001.tif]
