# Supplementary material for: Factors associated with the leftover rate of side dishes in Japanese school lunches
Source: PLoS One. 2024 Feb 26;19(2):e0298691. doi: 10.1371/journal.pone.0298691 (PMC10896521; doi:10.1371/journal.pone.0298691)
Supplement: Attachment — Submitted filename: PONE-D-23-10152 review.pdf [file pone.0298691.s006.pdf]

Decision: **Major Revision**.

Study of leftover food is the one of the best research area, because food waste is one of the major problem in the world in the point of economic, social and environmental. But, according to the following comments you should be corrected and revised the manuscripts.

Comment

- It is preferable to substitute "the study or the research" in instead of "we" in lines 26 and 29 of the abstract section on page 2.
- Under methodology part, line 113-114 what is your preference for selection of school/elementary?
- What are your preferences for choosing dates and months under the methodology component, lines 114–116? Why not choose the other months of the year? Also, why choose more dates for some months while doing the opposite for others. Why not use the same date, for instance, 3 for August and 21 for October?
- Please identify the standard reference for the formula used to compute the total weight of the leftovers under analysis items, lines 145–146. I believe it is not necessary to write the definition of leftover food under this heading, hence the caption "Definition of school lunch leftovers and the measurement method" needs to be changed.
- It is better to use "study area" or "sit of study" in place of "participant" in the methodology section. Additionally, it is preferable to use the caption "experimental design" to write the experimental design and procedure in a single paragraph.
- it is preferable to incorporate the information mentioned under "environmental factor" and "cooking factor" (independent variables) under "experimental design".
- Page 9, line 202, the caption "analysis method" should be changed by "data analysis method" because you were analyzing the experimental data under this.
- The table arrangement in your result part is unclear, therefore please rewrite the table in smart format.

- You ought to discuss the data in the table in relation to fact science (by citing the appropriate references) and compare it to earlier research that is related to this work. It is preferable to present the results in a table and discuss them at the same time.
- You must illustrate the impact of each independent component in the results section, including how they affected the amount of leftover food, both verbally and graphically. What happens when it comes to the leftover food for students as an independent element changes (increases and decreases).

Generally, under this work

- ✓ There is weak methodology (the experimental setup/design of the research, the methodologies for analysis, etc. are not written clearly).
- ✓ There is insufficient result discussion (the results you include in the table must be bolstered by an in-depth explanation, parallels, and references...)
- ✓ Lack of explanation or discussion of the impact of independent elements (cooking and environmental conditions) on the student's meal and how those factors affect the amount of leftover food?
