# Supplementary material for: Factors associated with the leftover rate of side dishes in Japanese school lunches
Source: PLoS One. 2024 Feb 26;19(2):e0298691. doi: 10.1371/journal.pone.0298691 (PMC10896521; doi:10.1371/journal.pone.0298691)
Supplement: Attachment — Submitted filename: Response_to_Editors_Reviewers.docx [file pone.0298691.s007.docx]

PONE-D-23-10152

Factors associated with the leftover rate of side dishes in Japanese school lunches

We would like to thank the Editor and Reviewers for their constructive critique to improve the manuscript. We have made every effort to address the issues raised and to respond to all comments. Please, find next a detailed, point-by-point response to the comments.

Response to Editor

1. Please ensure that your manuscript meets PLOS ONE's style requirements, including those for file naming. The PLOS ONE style templates can be found at

<https://journals.plos.org/plosone/s/file?id=wjVg/PLOSOne_formatting_sample_main_body.pdf> and

<https://journals.plos.org/plosone/s/file?id=ba62/PLOSOne_formatting_sample_title_authors_affiliations.pdf>

2. You indicated that ethical approval was not necessary for your study. We understand that the framework for ethical oversight requirements for studies of this type may differ depending on the setting and we would appreciate some further clarification regarding your research. Could you please provide further details on why your study is exempt from the need for approval and confirmation from your institutional review board or research ethics committee (e.g., in the form of a letter or email correspondence) that ethics review was not necessary for this study? Please include a copy of the correspondence as an ""Other"" file.

Response: According to the guidelines provided by the Research Ethics Committee of Okayama Prefectural University, ethics approval is not required for research projects that meet specific conditions. Firstly, if a project does not involve human subjects or data, materials of human origin, it is exempt from requiring an ethics application. Secondly, even if the project involves human subjects or identifiable data, it is still exempt if the information and data meet both of the following conditions:

- Data are existing and publicly available

- The subjects cannot be identified by any means

Please find attached the email exchange we had with the Ethics Committee Secretariat.

3. Please amend your current ethics statement to address the following concerns:

a) Did participants provide their written or verbal informed consent to participate in this study?

Response: No, they did not.

b) If consent was verbal, please explain i) why written consent was not obtained, ii) how you documented participant consent, and iii) whether the ethics committees/IRB approved this consent procedure.

Response: Not applicable.

4. We noted in your submission details that a portion of your manuscript may have been presented or published elsewhere. [Yes, I recently reported using the same data source in the Japanese article below.

A trial of food pattern analysis and leftover food evaluation of side dishes in a school lunch central kitchens in Okayama Prefecture. 7(1)72-80.2023,Bulletin of Higher Education and Liberal Arts and Sciences Research, Okayama Prefectural University doi/10.15009/00002459

However, in this paper, principal component analysis was performed from the weight of 17 food groups used in all canned side dishes, not just canned vegetables, to summarize the characteristics of school lunch side dish menus. The difference between this paper and the paper in preparation is the purpose (relationship of different factors such as environment, education, and cooking factors related to leftovers, targeting canned vegetables with a high rate of leftovers/establishment of a simple menu evaluation method). The method (principal component analysis/multiple regression analysis) is different. Two main factors were identified: ``Japanese ingredients'' (dried fish, root vegetables, seafood paste products) and ``main dish ingredients'' (fish and shellfish, meat). The result that the rate of leftover food is high in menus that use Japanese ingredients and/or fish as the main ingredient is different from the result of this paper.]

Please clarify whether this [conference proceeding or publication] was peer-reviewed and formally published. If this work was previously peer-reviewed and published, in the cover letter please provide the reason that this work does not constitute dual publication and should be included in the current manuscript

Response: As you pointed out, this paper diverges from the previous report in its primary objectives, methodology, and subject selection, as well as its outcomes. This manuscript is intended for a university-published bulletin and, as per the submission guidelines, is subject to peer review. In my cover letter, I articulated the reasons for including this study in the current manuscript, emphasizing that it does not amount to double publication.

5. We note that you have indicated that data from this study are available upon request. PLOS only allows data to be available upon request if there are legal or ethical restrictions on sharing data publicly. For more information on unacceptable data access restrictions, please see <http://journals.plos.org/plosone/s/data-availability#loc-unacceptable-data-access-restrictions>.

In your revised cover letter, please address the following prompts:

    a) If there are ethical or legal restrictions on sharing a de-identified data set, please explain them in detail (e.g., data contain potentially sensitive information, data are owned by a third-party organization, etc.) and who has imposed them (e.g., an ethics committee). Please also provide contact information for a data access committee, ethics committee, or other institutional body to which data requests may be sent.

Response: There are no ethical or legal restrictions. We request that our data be uploaded in the form of minimally anonymized data necessary to reproduce the results of our study.

   b) If there are no restrictions, please upload the minimal anonymized data set necessary to replicate your study findings as either Supporting Information files or to a stable, public repository and provide us with the relevant URLs, DOIs, or accession numbers. For a list of acceptable repositories, please see <http://journals.plos.org/plosone/s/data-availability#loc-recommended-repositories>.

We will update your Data Availability statement on your behalf to reflect the information you provide.

Response: SupportingInformation_suppl_data_23104 as a Supporting Information file.

Reviewer #1: Decision: Major Revision.

Thank you very much for your advice.
Response to Comment
¬ It is preferable to substitute "the study or the research" in instead of "we" in lines 26 and 29 of the abstract section on page 2.

Response: Thank you for your insightful review. I have made the correction as you suggested.

¬ Under methodology part, line 113-114 what is your preference for selection of school/elementary?

Response: We appreciate the reviewer's attention to detail. All schools served by communal Kitchen A were included in this study. No exclusions were made, and no specific selection criteria were applied for the choice of schools. Although the city of A contains other elementary and junior high schools with independent kitchens, we focused exclusively on schools served by Kitchen A. This approach enabled us to investigate the impact of food preparation, environmental factors, and educational approaches on leftover food, all based on side dishes originating from the same kitchen. It should be noted, however, that one limitation of our sampling is that fewer elementary schools than junior high schools were included among the target schools. This is primarily because, in the city of A, a larger percentage of elementary schools have their own kitchens.

¬ What are your preferences for choosing dates and months under the methodology component, lines 114–116? Why not choose the other months of the year? Also, why choose more dates for some months while doing the opposite for others. Why not use the same date, for instance, 3 for August and 21 for October?
¬ Please identify the standard reference for the formula used to compute the total weight of the leftovers under analysis items, lines 145–146. I believe it is not necessary to write the definition of leftover food under this heading, hence the caption "Definition of school lunch leftovers and the measurement method" needs to be changed.

Response: We appreciate the reviewer's attention to detail. To clarify, our analysis encompassed all school days in the months of May, June, July, August, September, October, and November. The aim was to examine the environmental factors affecting school lunch leftovers during periods that include both the hottest and most temperate times of the year, when maximum temperatures are at their peak. The number of school lunch days varies monthly, as lunches are not provided during school events and holidays. Additionally, there are fewer school lunches in August in Japan due to the extended vacation period.

¬ It is better to use "study area" or "sit of study" in place of "participant" in the methodology section. Additionally, it is preferable to use the caption "experimental design" to write the experimental design and procedure in a single paragraph.

Response: Thank you for these valuable suggestions. We have incorporated the official standard references and amended the section to "The Measurement Method of School Lunch Leftovers."

¬ it is preferable to incorporate the information mentioned under "environmental factor" and "cooking factor" (independent variables) under "experimental design".
¬ Page 9, line 202, the caption "analysis method" should be changed by "data analysis method" because you were analyzing the experimental data under this.

Response: Thank you for your valuable insights. I have opted for the caption "study area" in place of "participant," and have also utilized "experimental design" for further clarity.

¬ The table arrangement in your result part is unclear, therefore please rewrite the table in smart format.

Response: Thank you for your comment. We have revised the arrangement of the tables accordingly.

¬ You ought to discuss the data in the table in relation to fact science (by citing the appropriate references) and compare it to earlier research that is related to this work. It is preferable to present the results in a table and discuss them at the same time.

Response: Thank you to the reviewer for the valuable feedback. I have displayed the findings in Table 6 and included an additional explanation in the Discussion section.

¬ You must illustrate the impact of each independent component in the results section, including how they affected the amount of leftover food, both verbally and graphically. What happens when it comes to the leftover food for students as an independent element changes (increases and decreases).

Response: Thank you for the constructive suggestion. I have incorporated an illustrated Figure 1 into the Results section and have also added a corresponding explanation to the Discussion.

Generally, under this work
⎫ There is weak methodology (the experimental setup/design of the research, the methodologies for analysis, etc. are not written clearly).

Response: Thank you for your valuable advice. I have incorporated the experimental setup, study design, and analytical methods into the Methods section of the study.

⎫ There is insufficient result discussion (the results you include in the table must be bolstered by an in-depth explanation, parallels, and references...)

Response: Thank you for the insightful suggestion. I have included a table to elucidate the congruence between the results of this study and those of prior research, thereby highlighting the significance of the present investigation.

⎫ Lack of explanation or discussion of the impact of independent elements (cooking and environmental conditions) on the student's meal and how those factors affect the amount of leftover food?

Response: Thank you for the valuable suggestion. I have included an illustration to elucidate the influence of cooking and environmental factors on the leftovers of side dishes in school lunches.

Reviewer #2:

Thank you very much for your advice.
Response to Comment
1. The relevance of the manuscript must be clearly discussed- how does the factors associated with leftover rate of side dishes contributes to the nutrition intake of the school lunches. I perceived this article is part of a bigger research that was conducted and presenting the findings alone without the other parts of the work makes it incomplete. What exactly is the new knowledge that this manuscript is contributing to the existing knowledge in this field. Add a section on the new knowledge that this paper contributes to its audience- more like using an expository approach to dig deep into how these factors identified could improve the school lunch programs.

Response: We appreciate the reviewer's attention to detail. In this study, we collected data on the percentage of leftover vegetables in three different containers, as well as the types of ingredients used in each side dish, from this communal kitchen. However, the analysis only incorporated data on the leftover percentage of vegetables from May to November and data on the types of ingredients, seasoning, and cooking temperatures used in side dishes. Indeed, a more comprehensive approach would have included an assessment of how these leftover side dishes contributed to the nutrient content of the menu items served in the elementary and junior high schools associated with this kitchen. Therefore, we utilized available data from 40 representative days in June and November to ascertain the nutritional composition of the entire menu or individual side dishes, as well as the percentage of leftover canned vegetables in main dishes, side dishes, soups, and stews. These statistics are compiled in the accompanying tables. According to Table S-1, looking at the nutrient intake of side dishes after subtracting the leftover vegetables, the intake of dietary fiber, calcium, and iron, which are difficult to obtain even in daily home meals, is low even in school lunches, and the contribution of vegetable side dishes and soup leftovers is considered to be significant. As this does not constitute a complete data analysis, it limits our ability to discuss the study's impact comprehensively. Nonetheless, these figures serve as representative values for the warmer and more temperate months and are included for reference.

In addition, the new knowledge that this paper contributes to existing knowledge is highlighted around line 425-430.

2. Why focus on the leftover of the side dishes and not the main dishes? Is there an important message that the authors would like to convey to their readers about side dishes? The context of side dishes and its contribution to the overall nutrient intake of the population should be well articulated in the background section. The methodology of the manuscript should be revised to include what these side dishes, part of the side dishes that were always left over by the subjects and many other information that could provide the reader some context.

Response: Thank you for your review. The significance of vegetable intake, coupled with the fact that the nation's overall vegetable consumption is low—particularly among lower socioeconomic households—was highlighted in the background. Furthermore, it was emphasized that the inclusion of vegetable side dishes in school lunches helps to mitigate this disparity in children's diets.

Statistics pertaining to the leftovers of side dishes—as well as main dishes and soups or stews—in school lunches are provided in a supplemental table (SupportingInformation_fileS1). Additionally, a detailed account of the quantity and content of leftover side dishes has been incorporated into the Methods section. We extend our gratitude to the reviewer for highlighting the importance of these aspects.

3. Interested to know the content of the leftover and how much of these contributed to the food waste in the schools.

As shown in Supplemental table 1, food waste at schools is approximately 3.2 kg of main dishes, 11.8 kg of soup, and 6.0 kg of side dishes per day, with side dishes accounting for 28.5% of the remaining amount. Of these, soups include vegetables, but also include protein-based foods, meat and meat products and soup, and because of their high serving weight, they also have the highest weight of leftover vegetables. However, side dishes have the highest percentage of leftover vegetables, although they are served with less weight than soups and stews, and thus are considered to be the most representative of the tendency of leftover vegetable dishes. The main types and contents of vegetable side dishes that are often left and uneaten the content of the leftover shown in Supplemental table 2.

4. Add a section on the implication of the findings of this study.

Response: We have added the section to the discussion (pages 25, lines 425-430). We appreciate the reviewer's insightful comment.

5.The results section seems to have lengthy description of the tables - I suggest only relevant data should be described. The discussion of the results was inadequately completed- more work should be done to bring out clarity about the results and the relevance of this study.

Response: Thank you for your valuable advice. I have streamlined the description of the results to focus solely on pertinent data, and have also incorporated figures and tables to elucidate both the results and the study's relevance.
