# Supplementary material for: Factors associated with the leftover rate of side dishes in Japanese school lunches
Source: PLoS One. 2024 Feb 26;19(2):e0298691. doi: 10.1371/journal.pone.0298691 (PMC10896521; doi:10.1371/journal.pone.0298691)
Supplement: Attachment — Submitted filename: Response to Reviewers.docx [file pone.0298691.s008.docx]

PONE-D-23-10152

Factors associated with the leftover rate of side dishes in Japanese school lunches

We would like to thank the Editor and Reviewers for their constructive critique to improve the manuscript. We have made every effort to address the issues raised and to respond to all comments and suggestions. The revisions are indicated with red font in the revised manuscript. Please, find below a detailed, point-by-point response to the Editor’s and Reviewers’ comments, and we hope that our revisions will meet the Editor’s and Reviewers’ expectations.

**Point-by-point responses to Editor’s and Reviewers’ comments**

Response to Editor’s comments:

*Journal Requirements:

Please review your reference list to ensure that it is complete and correct. If you have cited papers that have been retracted, please include the rationale for doing so in the manuscript text, or remove these references and replace them with relevant current references. Any changes to the reference list should be mentioned in the rebuttal letter that accompanies your revised manuscript. If you need to cite a retracted article, indicate the article’s retracted status in the References list and also include a citation and full reference for the retraction notice.*

**Response**: Thank you for appraising us about the journal policies regarding Reference list. We have reviewed all references and replaced the URL of reference No. 15 with the most recent one (pasted below for your reference).

“15. Survey on school lunch implementation status. Ministry of Education, Culture, Sports, Science, and Technology; February 2019(in Japanese) [cited Dec 12, 2023]. Available from: https://warp.ndl.go.jp/info:ndljp/pid/11293659/www.mext.go.jp/b_menu/toukei/chousa05/kyuushoku/kekka/k_detail/__icsFiles/afieldfile/2019/02/26/1413836_001_001.pdf. (Heisei 30).”

**Response to Reviewers' comments:**

***Reviewer #2:*** *I thank the authors for sufficiently addressing the questions- the paper is now richer in content and clearly fits well with modern science.*

**Response**: We greatly appreciate your valuable comment and assessment of our work, Dr. Paul Eme.

***Reviewer #3:*** *Thank you for your valuable work. The study is interesting and you can see my suggestions below;
1- The School Lunch Implementation Standards may provide details for the ingredients of students’ meals however, they cannot persuade students to eat all meals on their dishes. Here we can talk about many different factors such as the eating habits of their parents, the eating habits of students or food preferences of students. So how did you evaluate these characteristics?*

**Response**: Thank you very much for your valuable comments and for introducing the papers. Certainly, there are many factors that may affect consumption of school lunches, including parents' eating habits, students' eating habits, and students' food preferences. As per your suggestion, we have elaborated on this aspect in our revised manuscript as follows:

In lines 445-462, we have mentioned the feedback comments from students and teachers on the days when there was most leftover vegetables which were considered in association with the warming time, but not to any specific preferences for the vegetables themselves. We have added the following text to improve the discussion section.

“On the other hand, the study by Donadini et al. [35], which recorded the leftovers of each child and vegetables and fish from the meals of preschool children showed the relationship between the amount of leftovers, preferences and familiarity. Izumi et al. [22] also investigated leftovers in Japanese school lunches through focus interviews with nutrition teachers at elementary schools in Tokyo, and found that the following factors help minimize food loss: (1) social norms for leftover meals, (2) menu planning to increase exposure to unfamiliar and/or disliked foods, (3) nutrition education in school curriculum, (4) teachers’ lunch time practice related to portion size and time management, and (5) engagement of student. The social norms that encourages not to leave any leftovers while having meals at home because it is wasteful suggests that parents' eating habits and food choices at home may influence students' food preferences and may lead to a particular pattern of leftover vegetable dishes. Since our study focused only on the percentage of leftover side dishes and examined the results of the school-by-school survey, we were not able to examine the factors involved in detail. However, schools provided daily voluntary inputs regarding vegetable preferences such as “I don't like the taste of fried komatsuna with jako.”, “Some people did not like the bitterness of the vegetables.”, “The student is uncomfortable with vegetables and seems to have more leftovers than others.”, “The radish salad was bitter and pungent." and “If shiitake mushrooms are not a favorite, students seem to leave the entire side dish.””

*2- To decide what are the main reasons for leftover rates/types of students these (school educational environment, outside physical environment, and cooking factors) cannot be the only factors. There are many other factors and using only defined factors is not enough.*

**Response**: Thank you for this comment. We have added the observations of Chu et al. (doi: [10.3390/su15075947](https://doi.org/10.3390/su15075947)) where they have analyzed the causal factors that result in school lunch waste, using a mixed-methodology approach to collect data on school lunches in high schools in Taiwan. They identified seven factors contributing to school meal waste.

Lines 463-468: “Chu et al. [36] reported on the factors that enable school lunch waste, using a mixed-methodology approach to collect data on school lunches in high schools in Taiwan, combining document analysis, direct weighing, observation, and semi-structured interviews.　They identified seven factors contributing to school meal waste: quality of meals, strict budget restrictions, tracking and feedback systems, unanticipated factors, partial meal behaviors, environmental awareness, and lack of initiatives to reduce food waste.”

We believe that the above factors may also be contributing to leftover school lunches, which in turn are contributing to school-wide food waste.

*3- You present the results with statistical evaluations, however, evaluated factors are not enough to obtain a final conclusion about the leftovers of students in junior and high schools. For palatabilities of children/adolescents, there are many studies with varied evaluation criteria, however, your criteria are not enough to get a final decision. You should broaden your criteria to reach a better conclusion for the study.*

**Response**: Thank you very much for your insightful comment. This study was conducted based on the measurement records of the ratio of leftover side dishes in each school, and did not examine factors among the consumers or operational factors that reduce the ratio of leftover vegetables, which we believe is insufficient as you have pointed out. We have added the following text to elaborate this point as follows:

Lines 468-474: “In order to reach a better conclusion, further study is needed with the following factors included as those related to leftover vegetables and other side dishes that are considered to have a high percentage of leftover dishes: preferences and selective eating behavior as individual factors, sex ratio as classroom or school factors, unforeseen factors such as absenteeism, food disposal policies and budget limitations as the operating school lunch programs. Future considerations include inclusion in the requirements for factor investigation of food waste management system of school lunch and integration with qualitative research methods.
*4- l think it will be better if you read these articles below and improve your discussion part as well;
***doi.org/10.3390/su15075947
***doi.org/10.1016/j.ijgfs.2022.100520*

**Response**: Thank you very much for this suggestion. We have included the above papers in our reference list together with comments in the discussion.

“35. Donadini G, Spigno G, Fumi MD, Porretta S. School lunch acceptance in pre-schoolers. Liking of meals, individual meal components and quantification of leftovers for vegetable and fish dishes in a real eating situation in Italy. Int J Gastronomy Food Sci. 2022;28:100520. doi: [10.1016/j.ijgfs.2022.100520](https://doi.org/10.1016/j.ijgfs.2022.100520).

36. Chu C, Chih C, Teng C. Food waste management: A case of Taiwanese high school food catering service. Sustainability. 2023;15(7):5947. doi: [10.3390/su15075947](https://doi.org/10.3390/su15075947).”

In addition to the above comments, all spelling and grammatical errors have been corrected.

We look forward to hearing from you in due time regarding our submission and to respond to any further questions and comments you may have.

Once again, we thank you very much for the comments and suggestions.
